# Molecular aspects of the exercise response and training adaptation in skeletal muscle^✰^

**DOI:** 10.1016/j.freeradbiomed.2024.07.026

**Published:** 2024-07-24

**Authors:** Regula Furrer, Christoph Handschin

**Affiliations:** Biozentrum, University of Basel, Spitalstrasse 41, 4056, Basel, Switzerland

**Keywords:** Skeletal muscle, Exercise, Transcriptional regulation, Training adaptation, Athletic performance

## Abstract

Skeletal muscle plasticity enables an enormous potential to adapt to various internal and external stimuli and perturbations. Most notably, changes in contractile activity evoke a massive remodeling of biochemical, meta-bolic and force-generating properties. In recent years, a large number of signals, sensors, regulators and effectors have been implicated in these adaptive processes. Nevertheless, our understanding of the molecular underpinnings of training adaptation remains rudimentary. Specifically, the mechanisms that underlie signal integration, output coordination, functional redundancy and other complex traits of muscle adaptation are unknown. In fact, it is even unclear how stimulus-dependent specification is brought about in endurance or resistance exercise. In this review, we will provide an overview on the events that describe the acute perturbations in single endurance and resistance exercise bouts. Furthermore, we will provide insights into the molecular principles of long-term training adaptation. Finally, current gaps in knowledge will be identified, and strategies for a multi-omic and –cellular analyses of the molecular mechanisms of skeletal muscle plasticity that are engaged in individual, acute exercise bouts and chronic training adaptation discussed.

## 1 Introduction

Locomotion is a fundamental function, with ancestral origin throughout the animal kingdom. A close co-selection of brain and skeletal muscle traits led to the evolution of human bipedal gait and persistence hunting. An intricate regulatory system thereby ensured plastic changes to either promote or limit muscle mass, matched to the prevailing environment, e.g. the availability of prey and food. In times of scarcity, energetically costly muscle mass was confined by the evolutionary selection for and maintenance of myostatin and other regulators that promote a reduction in muscle mass. Inversely, regular use of skeletal muscle for various tasks engages complex biological programs encoding adaptive processes to repeated cycles of contraction. In many societies, the need for physical activity has declined, while energetically rich food is available in abundance in the present day. This lifestyle-caused inadequacy in engaging skeletal muscle is a strong and independent risk factor for many chronic diseases [1]. Leisure time physical activity, even though “frivolous” in evolutionary terms due to the non-goal oriented tasks, e.g. acquisition of food, is an efficient intervention to restore contractile activity to normal levels, above the pathological baseline imposed by a sedentary lifestyle. Accordingly, regular physical exercise has tremendous health-enhancing benefits to prevent and treat more than 35 different pathological conditions of various etiologies and types [2,3]. Furthermore, being physically active reduces the risk of all-cause as well as disease-specific mortality (i.e. cardio-vascular disease or cancer) by 50–75 % [4]. Consequently, the World Health Organization (WHO) recommends a minimum engagement in physical activity to prevent numerous non-communicable diseases [5], even though the amount and best training modalities are still being debated [6]. Such uncertainties exist due to the relative scarcity of knowledge about the mechanistic underpinnings of muscle plasticity that hamper the design of evidence-based, individualized, safe and efficient training protocols.

In essence, a single bout of exercise is a stressor by disrupting cellular homeostasis. For comparison, many of the alterations elicited in this context resemble those seen in aging [7]. These potentially detrimental events are mitigated by normally inherently resilient muscle tissue plasticity, and eventually reversed when the perturbations are repeated over weeks, months and years. Such training elicits adaptive processes to further enhance the resilience of muscle and other tissues against these recurring challenges, thereby conferring functional improvement as well as performance and health benefits, a phenomenon termed “hormesis”. Thereby, molecular patterns are produced, which, at least in part, are in opposition to those observed in pathological settings. For example, the proteome of a trained muscle exhibits negative correlations with that of middle-aged individuals and those with impaired glucose tolerance [8]. Additionally, numerous genes altered in individuals with type 2 diabetes or metabolic syndrome show opposite regulation in athletes [9]. Strikingly, 6–12 months of training can substantially diminish the pathological transcriptomic fingerprint in these individuals [9]. Thus, the study of exercise physiology extends far beyond improving athletic performance. In modern societies in particular, an active lifestyle with high leisure-time physical activity would reflect the “normal” state of muscle engagement as selected for by evolution, and promotes the main physiological task of this tissue, the generation of different types of forces.

The morphological, cellular and metabolic adaptations that lead to functional improvements following training are well-described and show high specificity depending on the training modality, at least at the extremes of the spectrum [10]. For example, hallmarks of strength/power/sprint athletes include increased muscle mass and extraordinary power output over short periods of time. In comparison, endurance athletes are distinguished by augmented fatigue resistance and thus the ability to sustain muscle contractions for hours [10]. To evoke such divergent phenotypes, in a simplified manner, resistance training typically elicits a pronounced increase in muscle fiber size, while endurance training primarily induces mitochondrial biogenesis and metabolic remodeling, leading to an elevated oxidative capacity, without substantial effects on absolute muscle mass. Overall, the comparison of world-class athletes to the general population and patients suffering from muscle-associated diseases reveal the amazingly broad spectrum of human muscle functionality, adaptability and plasticity.

**Fig. 1:**
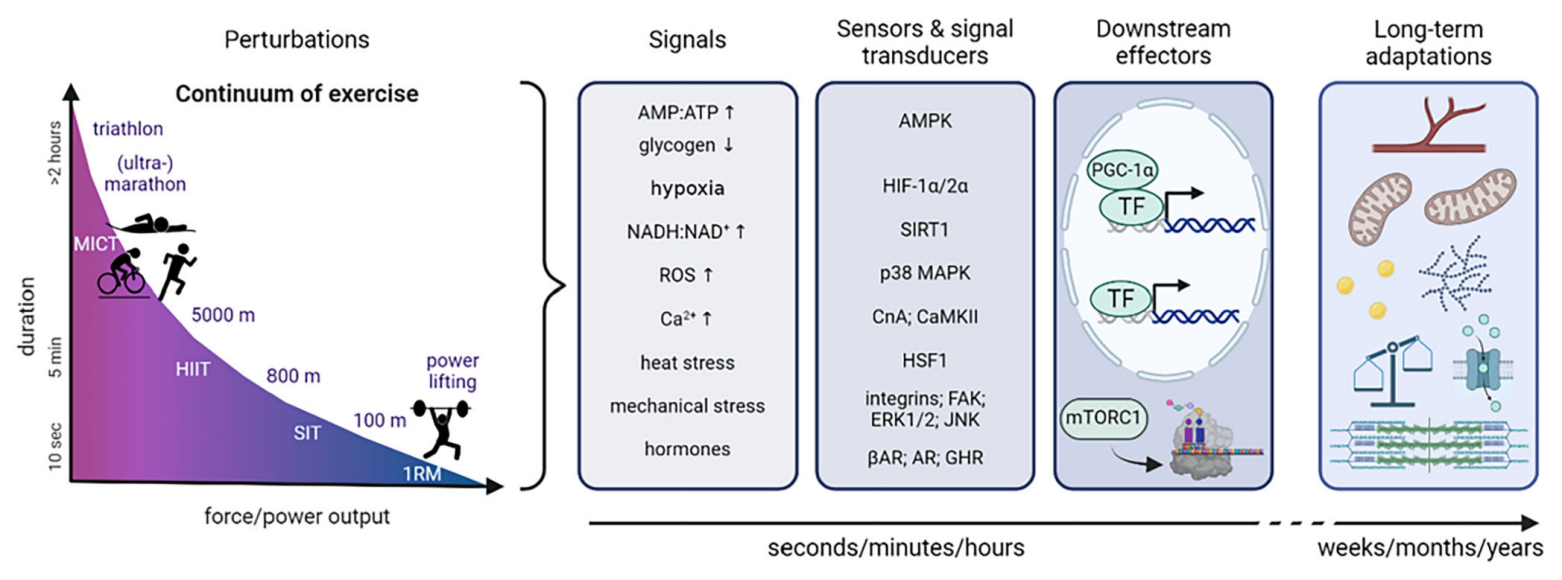
Continuum of exercise and divergent signals that contribute to training adaptation. While exercise paradigms are often divided into endurance or resistance exercise, there is a large overlap of these modalities in terms of power output, intensity or duration. The perturbations induced by this broad spectrum of types of exercise elicit various signals that subsequently are interpreted by various sensors. The molecular information of these acute signals is thereby transduced and converges on downstream effectors that control gene transcription, protein translation, proteolysis, enzymatic activities or other processes. If repeated over weeks or month, these acute perturbations will eventually result in training adaptation, characterized by increased vascularization, mitochondrial density, substrate uptake, utilization and storage, altered proteostasis, and elevated contractile elements. The latter is primarily observed in resistance-trained muscle, while the former processes are altered with endurance training. Abbreviations: MICT, moderate-intensity continuous training; HIIT, high-intensity interval training; SIT, sprint-interval training; 1RM, one repetition maximum; ROS, reactive oxygen species; AMPK, AMP-activated protein kinase; HIF-1α/2α, hypoxia-inducible factor 1α/2α; SIRT1, sirtuin 1; CnA, calcineurin A; CaMKII, Ca2+/calmodulin-activated kinase II; HSF1, heat shock factor 1; MAPK, mitogen-activated protein kinases; FAK, focal adhesion kinase; ERK1/2, extracellular-regulated kinase 1/2; JNK, c-jun NH2-terminal kinase; βAR, β-adrenergic receptor; AR, androgen receptor; GHR, growth hormone receptor; PGC-1α, peroxisome proliferator-activated receptor γ coactivator 1α; TF, transcription factor; mTORC1, mammalian target of rapamycin complex 1. Created with BioRender.com; sports icons from Adobe Stock, with permission.

It is noteworthy that between the extremes, e.g. power lifting vs. marathon running, many athletic disciplines require both strength and endurance (Fig. 1). Moreover, specific plastic changes might be triggered in a context-dependent manner. For example, endurance and resistance exercise can also increase muscle mass and oxidative capacity, respectively, in untrained individuals [11,12]. Thus, the differentiation between adaptations to endurance and resistance training can be somewhat arbitrary, and a substantial overlap in consequences observed. Accordingly, both training modalities are described by the general principles of duration (how long), frequency (how often), intensity (how hard) and specificity (general vs. discipline-specific). Training is organized in micro- (7–21 days), meso- (3–8 weeks) and macrocycles (1 season or year), in which these principles are varied to achieve a performance peak at the time of competition. Even though training for strength/power and endurance disciplines has these descriptors in common, distinct performance parameters are assessed for design and testing. For example, endurance exercise is primarily defined by intensity, typically expressed as a percentage of maximal oxygen uptake $\left(\dot{V}\;O_{2max}\right)$, maximal heart rate (HF_max_), peak power output or fractional utilization (i.e. lactate threshold or critical power), by volume, encompassing training duration as well as frequency, and by specificity. Consequently, endurance exercise can range from low- and moderate-intensity continuous exercise to high-intensity interval training (HIIT), typically performed at intensities exceeding 80 % HF_max_, or sprint-interval training (SIT), which generally involves all-out or supramaximal efforts [13]. Resistance exercise is determined by the intensity, often represented by the load expressed as percentage of one repetition maximum (1RM) and duration, frequency and specificity, sometimes combined with descriptors of frequency of training of a specific muscle group, number of repetitions and sets, as well as time under tension. Additional parameters include the type of contraction (concentric/myometric/shortening, isometric or eccentric/plyometric/lengthening), along with contraction velocity (determining power) and rest periods between sets. Therefore, the specific execution and resulting response can substantially vary even within each of the two paradigms and in many disciplines, a mixed form or combination of resistance and endurance training is aimed for. Of note, the vast possibilities of changing the aforementioned training descriptors can contribute to discrepancies in the literature in regard to outcome and the molecular response if generalized conclusions are drawn. Nevertheless, to simplify the discussion, we will not discriminate between the various subtypes of exercise and will use the broad categories of submaximal and maximal/all-out endurance or resistance exercise in this review.

Despite the striking and very distinct adaptations to endurance and resistance exercise as evidenced by the muscle characteristics of endurance and strength/power athletes, the molecular mechanisms underlying the specificity of training adaptation remain largely elusive. In this review, we will provide an overview of some of the known signaling pathways that are engaged during an acute bout of exercise and discuss the current molecular principles of training adaptation. In addition, the molecular response of an untrained and trained muscle will be reviewed as well as the effects of exercise and training on the multicellular response in muscle.

## 2 Molecular mechanisms during acute exercise

The molecular response of skeletal muscle tissue to exercise is defined by of a broad range of signals, originating from outside and inside the tissue, which ultimately drive physiological adaptations (Fig. 1). Amongst others, these signals include mechanical stress, changes in substrate and oxygen availability, circulating hormones as well as alterations in intracellular calcium concentrations ([Ca^2+^]i), metabolic and oxidative stress and a rise in muscle temperature [10]. Sensor proteins such as receptors, kinases and phosphatases transduce molecular information and initiate complex molecular cascades involving post-translational modifications and protein-protein interactions. Ultimately, these cellular signals culminate in the activation of regulators that control gene transcription, protein synthesis, proteolysis or other enzymatic activities. In the past decades, the combined efforts of researchers to shed light on the complex regulatory network lead to a large number of factors described to be involved in training adaptation [10,14,15]. Key factors have emerged, including the mammalian target of rapamycin complex 1 (mTORC1), AMP-activated protein kinase (AMPK) or the peroxisome proliferator-activated receptor (PPAR) γ coactivator 1α (PGC-1α). Accordingly, these proteins, and the expression of the respective genes, are frequently studied to evaluate the effects of an exercise intervention. AMPK protein activation and PGC-1α gene transcription, along with other target genes involved in metabolic processes, are commonly used as indicators of a successful response to an endurance exercise bout. In contrast, mTORC1 activation and downstream signaling are often assessed to understand the impact of resistance exercise on muscle protein synthesis and fiber hypertrophy. It however is important to note that the historic use of these and other putative “marker” factors most likely is inadequate, for several reasons. First, current technologies allowing a more comprehensive and unbiased assessment of the acute exercise response have produced a long list of potential mediators and regulators of exercise-mediated muscle plasticity, important for signal integration and output coordination in a complex network [16–19]. Thus, the current focus on the “usual suspects” reinforces an oversimplified picture and fails to take into account the physiological and mechanistic complexity. Second, mTORC1, AMPK, PGC-1α and most of the other factors that have been identified to date are only activated in a short-term manner, e.g. by acute changes in energy substrate availability, and their relevance for long-term adaptations is unclear. Third, most of the cellular perturbations are observed in endurance and resistance training, for example mechanical stress or [Ca^2+^]i. Accordingly, even though assessed for distinct adaptations, many of the sensor and effector proteins, including mTORC1, AMPK and PGC-1α are activated by both training modalities, and the mechanisms of specification of the response is unknown [20,21]. Fourth, the mechanistic network that integrates various signals and coordinates adaptations most likely is far more complex than currently appreciated, consisting of redundancies and alternate pathways that ensure plastic changes even in unfavorable conditions. For example, even though muscle PGC-1α is indispensable for a normal response of the epigenome, transcriptome, proteome and phosphoproteome to an acute bout of exercise or chronic endurance training, mice with a specific ablation of *Ppargc1a*, the gene encoding PGC-1α, improve performance with training, albeit at a much lower level compared to wildtype littermates [22]. This training effect manifests despite a significant dampening of metabolic re-wiring, e.g. related to lactate or ketone body homeostasis, tissue vascularization or change in $\dot{V}\;O_{2max}$ [22–25]. Similar observations have been made for other factors, including AMPK or mTORC1, indicating that there might not be a single “master regulator” for exercise adaptation in muscle [10]. In the following sections, we discuss some of these processes and summarize evidence for involvement in different training paradigms. For a more complete overview on known perturbations and molecular mechanisms in exercise adaptation in skeletal muscle, the reader is referred to other contemporary reviews [10,15].

### 2.1 Extracellular signals during exercise

**Fig. 2:**
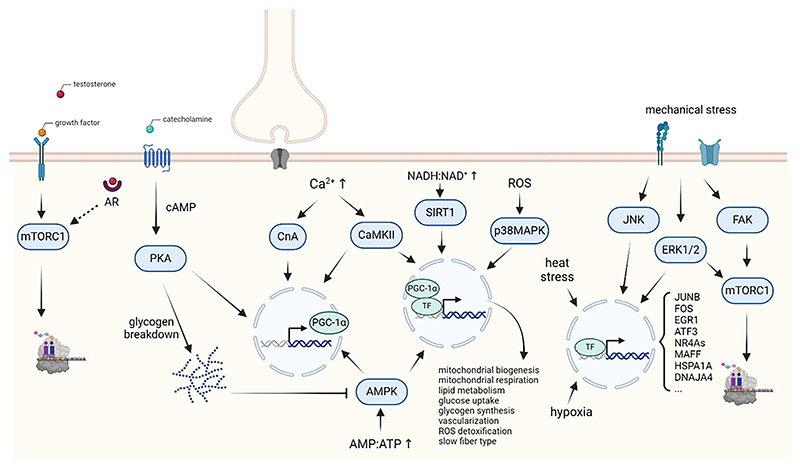
Signaling pathways activated during an acute bout of exercise. Examples of extra- and intracellular signals that are induced during endurance and resistance exercise, leading to downstream effects that help to cope with the different types of stress during an exercise bout, and initiate processes for a long-term increase in resilience and functional adaptation. Abbreviations: AR, androgen receptor; mTORC1, mammalian target of rapamycin complex 1; cAMP, cyclic AMP; PKA, protein kinase A; CnA, calcineurin A; CaMKII, Ca2+/calmodulin-activated kinase II; PGC-1α, peroxisome proliferator-activated receptor γ coactivator 1α; TF, transcription factor; AMPK, AMP-activated protein kinase; SIRT1, sirtuin 1; ROS, reactive oxygen species; MAPK, mitogen-activated protein kinases; JNK, c-jun NH2-terminal kinase; ERK1/2, extracellular-regulated kinase 1/2; FAK, focal adhesion kinase; JUNB, JunB proto-oncogene; FOS, Fos proto-oncogene; EGR1, early growth response 1; ATF3, activating transcription factor 3; NR4A, nuclear receptor subfamily 4 group A; MAFF, MAF BZIP transcription factor F; HSPA1A, heat shock protein family A (Hsp70) member 1A; DNAJA4, DnaJ heat shock protein family (Hsp40) member A4. Created with BioRender.com.

In anticipation and at the onset of exercise, the release of catecholamines prepares the body for the physiological demands of exercise, analogous to the fight-or-flight response. Thereby, systemic and local adaptations are initiated, including increased heart rate, breathing frequency and energy substrate mobilization from adipose tissue and liver by promoting lipolysis and hepatic glucose output (through glucogenolysis and gluconeogenesis), respectively, geared towards efficient muscle contractility to boost performance [26]. In skeletal muscle, agonist binding to β_2_-adrenergic receptors leads to the activation of different signaling pathways such as the cyclic AMP (cAMP)-protein kinase A (PKA) axis (Fig. 2). Thereby, various processes are initiated in muscle fibers to support contraction, including enhanced glycogen breakdown via glycogen phosphorylase activation [10]. Of note, β_2_-adrenergic stimulation also results in transcriptional regulation, e.g. of PGC-1α expression [27]. Elevated levels of catecholamines are observed both in endurance and resistance exercise [28]. Stress-related pathways, e.g. the hypothalamic-pituitary-adrenal axis or cortisol, exhibit distinct engagement in the anticipatory phase compared to peri-exercise and depend on the training state [28]. Furthermore, cortisol levels are influenced by exercise intensity, and differ between training paradigms, being higher after a single bout of endurance compared to resistance exercise [28]. The regulatory and functional consequences of these differences are however poorly understood.

During exercise, these and many other circulating factors, originating from various tissues, often collectively referred to as exerkines, affect muscle performance [29]. For example, the levels of growth hormone (GH), insulin-like growth factor 1 (IGF-1) and testosterone are all increased in response to exercise. Besides the stimulation of IGF-1 production in the liver, GH also promotes anabolic signaling within muscle [15]. However, the exact mechanism remains unclear [28]. Furthermore, IGF-1, and its splice isoform mechano-growth factor (MGF), are also synthesized in contracting muscle, exerting auto- and paracrine anabolic effects, to a large extent by activating the IGF-1 receptor, thereby stimulating the insulin receptor substrate (IRS)-phosphoinositide 3-kinase (PI3K) signaling pathway, resulting in activation of protein kinase B (PKB/Akt) and mTORC1 [15]. The ensuing stimulation of protein synthesis, together with the Akt-FoxO-mediated repression of protein degradation results in a shift in the balance of muscle proteostasis towards an increased muscle protein synthesis rate. Similar to GH and IGF-1, testosterone promotes anabolic pathways through its binding to the androgen receptor (Fig. 2) [30]. The augmentation of testosterone is observed in both men and women [15, 31–33]. Despite the association of these hormones with anabolic signaling, both resistance and endurance exercise can increase circulating levels, with the duration and intensity determining the extent of elevation [15]. Higher loads, shorter rest periods and greater volume tend to result in more pronounced rise in GH levels, reaching 300–500 % of basal levels [15,28]. Of note, enhanced levels can persist for up to 3 h [28]. Whether and how these extracellular signals contribute to training specificity thus remains elusive.

### 2.2 Ca^2+^ signaling and fiber type specification

The primary consequence of action potential transmission at the neuromuscular junction is the increase in [Ca^2+^]i, through a process called excitation-contraction coupling. Muscle contractions are enabled by binding of Ca^2+^ to troponin C, a pre-requisite for the interaction between myosin and actin filaments. Consequently, there is an elevated Ca^2+^ flux during muscle contraction. In addition, Ca^2+^ is involved in many signaling pathways and cellular processes in the muscle cell. Binding to calmodulin, and the ensuing activation of the Ca^2+^/calmodulin-activated kinase II (CaMKII) and the phosphatase calcineurin A (CnA) modulates various downstream targets (Fig. 2). For example, CnA phosphorylates and thereby activates the transcription factor nuclear factor of activated T cells (NFAT), initiating its translocation to the nucleus and promoting the expression of the oxidative/slow-twitch muscle fiber-typical gene program [34]. This transcriptional change is supported by CnA and CaMKII-mediated engagement of the myocyte enhancer factor 2 (MEF2), which in turn, amongst other target genes, induces transcription of PGC-1α, and thereby also an oxidative/slow-twitch muscle fiber program [10]. During prolonged low-intensity exercise, Ca^2+^-mediated engagement of CaMK kinase β (CaMKKβ) results in higher AMPK activity even in the absence of an energetic deficit, which typically is the primary trigger for AMPK activation [35]. CaMKII and AMPK promote the expression of PGC-1α by enhancing the transcriptional activity of the cAMP-response element binding protein (CREB) and activating transcription factor 2 (ATF2) [10, 36,37]. Even though most of these signaling pathways have been associated with endurance exercise and an oxidative/slow-twitch muscle fiber specification, [Ca^2+^]i obviously rises in all types of muscle contractions. The amplitude and duration of [Ca^2+^]i may vary depending on the exercise modality and fiber type. The peak amplitude of [Ca^2+^]i during muscle contraction is substantially higher in glycolytic/fast-twitch type II fibers compared to oxidative/slow-twitch type I fibers [38,39]. Interestingly, NFAT translocation to the nucleus is predominantly induced by low-frequency rather than high-frequency bursts, the latter resembling resistance- or sprint-type exercise [40]. It however is unclear how the specification of [Ca^2+^]i and downstream activation of effectors such as CaMKII and CnA is encoded in glycolytic/fast-twitch and oxidative/slow-twitch, and resistance and endurance training, respectively, as these regulators are engaged in both modalities [41], and pharmacological inhibition of CnA also affects muscle hypertrophy in various animal models [42]. Importantly, robust muscle fiber-type changes only occur in long-term training adaptation, and not after single acute exercise bouts. Thus, the mechanisms that integrate the transient gene expression changes into persistent adaptations in fiber type remain to be elucidated. Of note, shifts in muscle fiber type distribution in most cases occur in similar fashion in both training modalities, leading to a lower relative abundance of glycolytic/fast-twitch type IIX fibers [43–46]. Nevertheless, endurance and strength/power elite athletes exhibit opposing overall distributions with high slow-twitch in the former, and more numerous fast-twitch muscle fibers in the latter [10,47–49]. The extent to which these distributions are determined by training adaptations, genetic endowment and other pre-determining factors is unknown. Curiously, in transgenic models that overexpress PGC-1α [50] or activated forms of CnA [51] and CaMK [52] in skeletal muscle, the shift towards oxidative/slow-twitch muscle fibers seems capped at around 15–30 % in relative distribution. It is mysterious why only some muscle fibers shift in terms of contractile properties, even though these factors are constitutively expressed in all fibers. However, a much more pronounced shift in oxidative metabolism is observed, e.g. measured by succinate dehydrogenase activity, indicating that metabolic and contractile fiber types can be separated [53].

### 2.3 Mechanosensing

Repeated muscle contractions exert considerable mechanical forces on various cellular structures, including the extracellular matrix (ECM), plasma membrane, cytoskeleton and the nuclei. These forces, comprising compression, stretch, and shear stress, are accordingly sensed by various molecules, such as mechanically activated ion channels, integrins and integrin-associated proteins [54]. Mechanical stress leads to an influx of Ca^2+^, which can trigger Ca^2+^-dependent pathways. Additionally, many of these mechanical signals converge on mTORC1, implying a close association with resistance exercise and hypertrophy (Fig. 2). Moreover, fully functional mechanically activated ion channels are essential for mTORC1 activation in response to eccentric contractions [54]. Likewise, focal adhesion kinase (FAK) interacts with integrins, contributing to mechanotransduction through the activation of mTORC1-p70S6K pathway [54,55]. Furthermore, the ζ isoform of diacylglycerol kinases (DGKs), located in membranous structures, has been implicated in the stretch-dependent activation of mTORC1 [56]. Interestingly, prolonged periods of unloading induce “mechano-anabolic resistance”, characterized by reduced phosphorylation of the mTORC1 downstream signaling effector p70S6K after exercise [54]. Despite these links, the exact mechanisms underlying mTORC1 activation remain unclear. Other signaling pathways might bypass mTORC1. For example, the mitogen-activated protein kinases (MAPK), including extracellular-regulated kinase 1/2 (ERK1/2), c-jun NH2-terminal kinase (JNK) and p38 MAPK are engaged in mechanotransduction [10]. *In situ* experiments revealed tension-dependent phosphorylation of ERK1/2 and JNK, with the most pronounced effects observed after eccentric contraction followed by isometric, concentric and passive stretch [57]. This could imply that these MAPKs may contribute to contraction-induced differences in downstream signaling. Thus, the mechanisms controlling specification of mechanosensing in different types of exercise are murky. For example, although ERK1/2 signaling is typically linked to an increase in protein synthesis, elevated ERK1/2 phosphorylation has been reported after endurance, sprint and resistance exercise [16]. Similarly, FAK phosphorylation was boosted after both endurance and resistance exercise and does not seem to be affected by training history [21]. In line with these observations, mTORC1 and p70S6K phosphorylation is elevated following endurance and resistance exercise [21,58]. It thus seems that despite the differences in forces that impinge on muscle fibers, many mechanosensing pathways are engaged in endurance and resistance exercise, including those initiated by sensors in sarcomeric, cytoskeletal and nuclear structures [54]. How the muscle cell differentiates between short bouts of high force/load- and enduring (sometimes for hours) low force-induced mechanical stress in resistance and endurance exercise bouts, respectively, is unknown.

### 2.4 Oxidative stress

Prolonged muscle contractions increase the production of reactive oxygen species (ROS) and reactive nitrogen species (RNS). The resulting oxidative stress plays a critical role in training adaptation [59]. The free radicals superoxide $\left({\text{O}}_{2}^{.-}\right)$, derived from a one-electron reduction of molecular O_2_, and nitric oxide (NO), produced by the conversion of L-arginine by the NO synthase (NOS), are the parent molecules of ROS and RNS, respectively [60]. In contracting muscle fibers, neuronal NOS (nNOS) is the main contributor of NO. The NADPH oxidase NOX2 is the primary source of ROS during muscle contraction, while NOX4 mainly contributes to the production of basal ROS [10,59]. Furthermore, elevated [Ca^2+^]i promotes the cleavage of membrane phospholipids by phospholipase A2 (PLA2) and the concomitant release of arachidonic acid that triggers ROS-producing enzymes such as lipoxygenases and NOX2 [10,60]. Mitochondria play a less prominent role for ROS production in the muscle fiber during exercise. The pronounced increase in ROS production during exercise is intensity- and duration-dependent and appears to be affected by the intrinsic muscle temperature [59]. An optimal ROS concentration in muscle, which is the result of the integration of ROS production and scavenging by antioxidants, is required for maximal force generation [59]. However, the exact mechanisms of the influence of ROS on force production remain elusive. In addition to the acute effects on force generation, ROS/RNS are engaging various signaling pathways. For example, ROS activate p38 MAPK, ERK1/2 and JNK and downstream transcriptional targets [10,60]. Moreover, ROS promote the release of nuclear factor erythroid 2-related factor 2 (NRF2, encoded by the *NFE2L2* gene) from the redox-sensitive Kelch-like ECH-associated protein 1 (KEAP1) and thereby prevent NRF2 protein degradation [10]. Nuclear translocation of NRF2 and the binding to antioxidant response elements (AREs) boosts gene expression of components of the antioxidant system [10]. In addition, transcription of nuclear respiratory factor 1 (NRF1) and PGC-1α are induced by NRF2, implying a role of ROS in the regulation of oxidative metabolism and substrate utilization. Besides NRF2, ROS promote the transcriptional activity of nuclear factor κB (NF-κB), heat shock factor 1 (HSF1) and activator protein-1 (AP-1) [10,60]. While NF-κB also contributes to the transcription of antioxidant enzymes, HSF1 and AP-1 predominantly trigger a general stress and cytoprotective response [10,60]. Hence, the initial response of elevated ROS comprises an elevation of the antioxidant defense system and various other proteins involved in the protection against cellular damage. Consequently, regular exercise training leads to an increase in antioxidant enzymes and thereby promotes the resilience of the muscle against oxidative stress-induced cellular damage [10,59]. In addition to the cytoprotective and metabolic effects, ROS also activates the Akt-mTORC1 signaling pathway and may thereby play a role in muscle fiber hypertrophy [10]. Therefore, ROS-induced signaling pathways are instrumental for both endurance and resistance training adaptation. Accordingly, both training paradigms result in an elevation of antioxidant enzymes [61].

### 2.5 Metabolic signaling

The high metabolic demand imposed by exercise results in an elevation of cellular ADP:ATP and AMP:ATP ratio. While ATP inhibits AMPK activity, the association of AMP to the γ subunit of AMPK leads to the phosphorylation and activation of AMPK by the upstream liver kinase B1 (LKB1) [35]. In contrast, glycogen binding to the β subunits inhibits AMPK activity (Fig. 2). Therefore, when glycogen levels are reduced during prolonged exercise, these inhibitory effects decrease, sensitizing and boosting AMPK activity [35]. AMPK orchestrates processes to facilitate ATP generation and suppresses anabolic pathways [35]. For example, AMPK promotes the uptake and utilization of glucose and fatty acids, and increases protein degradation via autophagy and the ubiquitin-proteasome pathway [35,62]. Moreover, catabolic processes and ATP generation are promoted by AMPK via phosphorylation and transcriptional induction of PGC-1α [62]. In contrast, AMPK confines energetically demanding anabolism, in particular protein and glycogen synthesis via inhibitory effects on mTORC1 and glycogen synthase, respectively [35]. The inhibition of mTORC1 by AMPK has been postulated as a regulatory nexus to shift from anabolism to catabolism, representing one of the molecular explanation for a potential training interference in which one training modality, i.e. endurance training resulting in AMPK activation, reduces the effect of a different paradigm, i.e. resistance training depending on mTORC1 activation for muscle protein synthesis and fiber hypertrophy. For example, high- and low-frequency stimulation of isolated rat muscle has been demonstrated to specifically activate the Akt-mTORC1 or AMPK-PGC-1α pathways, respectively [63]. Assuming that such stimulations are a model for resistance and endurance exercise, respectively, the authors proposed the existence of an “AMPK-Akt master switch” that may determine training specificity [63]. However, the response to *bona fide in vivo* exercise appears to be much more complex. While AMPK is typically activated during exercise at intensities of at least 60 % $\dot{V}\;O_{2max}$ [35], AMPK engagement has also been observed during exercise at very low intensities (30–40 % $\dot{V}\;O_{2max}$ if performed to exhaustion), upon HIIT and even after resistance exercise [15,16,35,64]. In fact, phosphorylation of mTORC1, p70S6K, and AMPK is simultaneously elevated after both endurance and resistance exercise, independent of training status [21]. It thus is unclear how specification is brought about, e.g. the divergence in mitochondrial and myofibrillar fractional synthetic rates. The former is enhanced by resistance and endurance exercise in untrained, but only by endurance exercise results in trained individuals [21]. Conversely, myofibrillar fractional synthetic rate is only augmented in response to resistance exercise [21]. This flexibility of activation of AMPK, mTORC1 and other regulators in endurance and resistance training could provide an explanation for the overall minimal extent of interference in concurrent training that is observed humans, at least on the recreational level [11,65]. Moreover, the role of these regulators in controlling catabolism and anabolism might not be as strict as initially postulated. For example, in the right context, AMPK can enhance skeletal muscle anabolic capacity and promote cell survival [66]. Collectively, these examples illustrate the poor knowledge about the molecular mechanisms that control exercise adaptations, in particular related to specification of cellular, morphological and functional changes evoked by different training paradigms.

### 2.6 Transcriptional response to an acute exercise bout

**Fig. 3:**
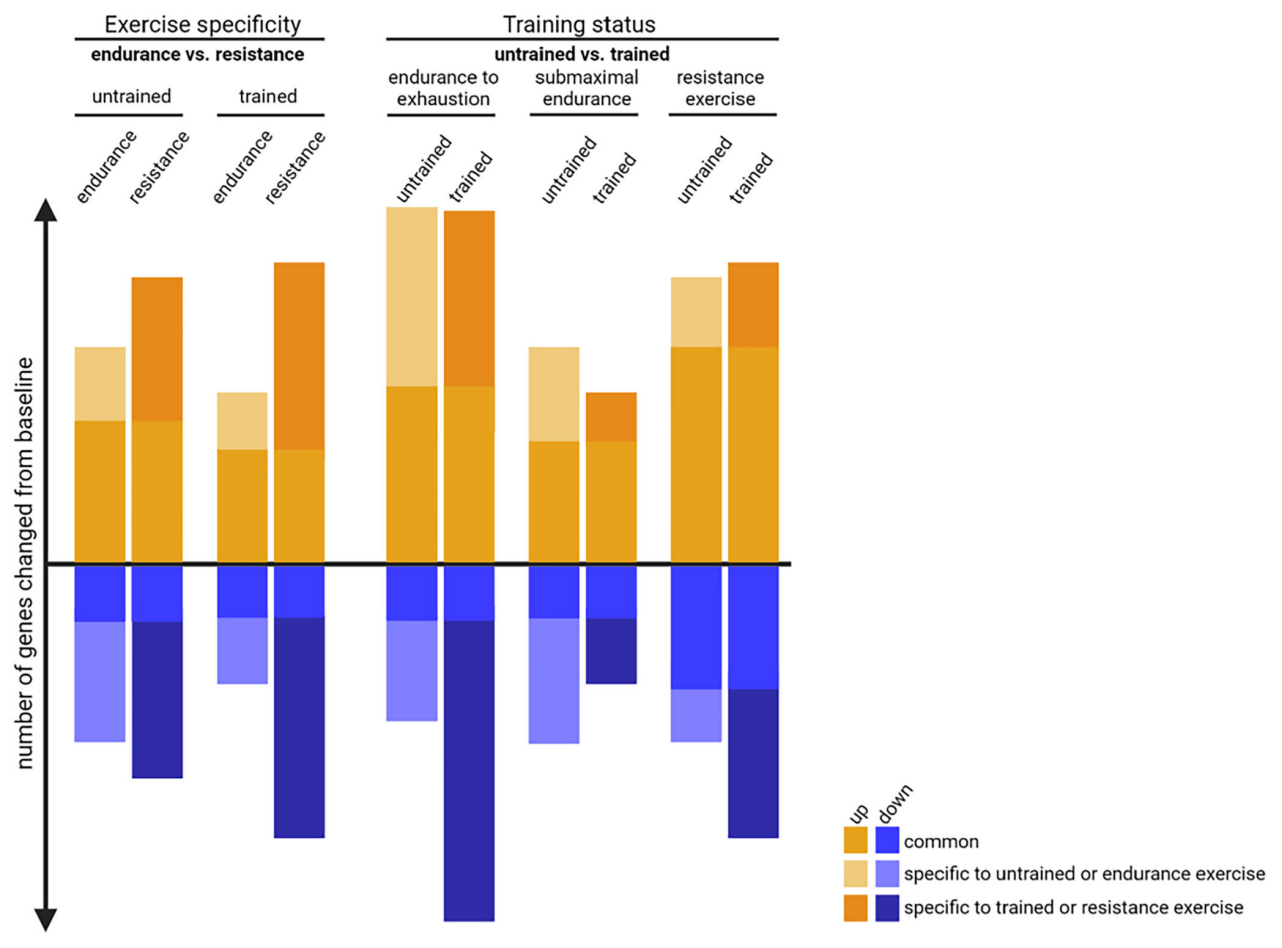
Common and specific transcriptional responses to an acute challenge. Visualization of the common and unique response to an acute exercise bout depending on exercise modality (comparing endurance and resistance exercise) or training state (comparing untrained and trained muscle). Each context exhibits a common as well as specific response. Surprisingly, endurance and resistance exercise share a large number of regulated genes, with a more pronounced specification in resistance compared to endurance exercise regardless of the training state. The proportion of commonly regulated genes is higher in submaximal endurance exercise and resistance exercise, while endurance exercise to exhaustion results in a higher specification of the response of a trained compared to an untrained muscle. Finally, in almost all of these contexts, the specification of the downregulated surpasses that of the upregulated genes. Data from Refs. [20,22]. Created with BioRender.com.

Despite the very similar signals and pathways elicited by endurance and resistance exercise, a single bout of resistance exercise induces larger changes in gene expression compared to an acute bout of endurance exercise (Fig. 3) [20,67]. The higher number of transcripts altered in response to resistance exercise implies a greater level of perturbation. One potential explanation for the increased number of genes altered after resistance exercise may be attributed to the eccentric component of the exercise, which could exert greater stress on the muscle and consequently lead to a more prominent transcriptional response. Consistent with this notion, downhill running, which involves pronounced eccentric contractions, promotes the transcription of at least twice as many genes 4 h post-exercise compared to uphill running, which primarily involves concentric contractions [68]. In addition, there is some evidence that the nature of contraction during resistance exercise differentially modulates the muscle proteome [69]. Therefore, the type of contraction applied during exercise appears to substantially impact the transcriptional response.

A plethora of factors have been implicated in the exercise response of skeletal muscle (Fig. 2) and a substantial number of sensor protein-induced signaling converges on the transcription and activation of PGC-1α [10,70–72]. PGC-1α is a regulatory nexus for endurance training adaptation and orchestrates broad gene programs by coactivating various transcription factors [70,73,74]. For example, coactivation of NRF1 and NRF2 (encoded by the GA-binding protein (*GABP*) gene) promotes the transcription of nuclear-encoded mitochondrial genes as well as the mitochondrial transcription factor (TFAM). Subsequently, TFAM translocates to the mitochondria to control the transcription of mitochondrial-encoded genes [73]. Other interaction partners of PGC-1α include the estrogen-related receptor α (ERRα), PPARs, MEF2 and NRF2/NFE2L2 to coordinate the expression of genes involved in mitochondrial biogenesis, angiogenesis, fatty acid oxidation, glucose uptake, fiber type determination, neuromuscular junction and ROS detoxification [10,71,73]. In fact, elevation of PGC-1α in skeletal muscle is sufficient to induce an endurance-trained phenotype [50,75], while specific gene ablation provokes detrimental outcomes associated with pathological inactivity [23,76] as well as non-physiological training adaptation [22,27,77]. The transcriptional regulation of muscle PGC-1α appears to be exercise intensity-dependent, as evidenced by the 10-fold increase 3 h after high-intensity exercise $\left(80\%\;\text{of}\;\dot{V}\;O_{2max}\right)$ as opposed to the 4-fold elevation after low-intensity exercise $\left(40\%\;\text{of}\;\dot{V}\;O_{2max}\right)$ [78]. With training progression or in athletes, the induction of PGC-1α is reduced after exercise at the same relative intensity in some [20,79], but exacerbated in other studies [80]. Although PGC-1α is primarily linked to endurance training adaptation, resistance exercise also triggers a pronounced increase in PGC-1α expression [20,67]. It is not clear whether divergence in functional outcome is mediated by different isoforms of PGC-1α [81]. In many ways, PGC-1α activity represents an acute stress response, for example in the context of mismatched energetic demand and availability. The robust, immediate, and relatively short-lived transcriptional induction of muscle PGC-1α during and after an exercise bout thus is similar to that of a number of immediate early genes, such as JUNB, FOS, EGR1, ATF3, MAFF and NR4As, along with genes related to heat stress such as HSPA1A, DNAJA4, which all exhibit a high induction in both exercise paradigms [18,20,67]. In fact, approximately 75 % of the genes upregulated after a single bout of endurance exercise are also altered following resistance exercise alluding to a general stress response [20,67]. Since resistance exercise elicits more transcriptional changes, a larger number of gene are specific to the resistance paradigm (Fig. 3). Notably, even though often underappreciated, a considerable number of genes are not elevated, but reduced after an acute challenge [82]. For example, all-out cycle sprints or resistance exercise to failure in active or trained individuals, respectively, resulted in a comparable number of genes that were increased or decreased post-exercise [82,83]. Strikingly, the suppression of genes seems to be more specific to the training paradigm. In fact, only around 40 % of the downregulated genes after a single bout of endurance exercise are also regulated after resistance exercise and therefore 60 % are specific to the endurance paradigm (Fig. 3) [20,67].

In summary, even though different stimuli are experienced in endurance or resistance exercise, the acute response, at least in an untrained muscle, exhibits a marked overlap, characterized to a large extent by a general stress response, e.g. caused by mechanical, energetic, redox, heat, proteostatic or oxygen perturbations [10]. In contrast to the relatively large number of commonly upregulated transcripts, the suppression of genes post-exercise appears to be controlled in a more targeted manner and exhibits a greater specificity to the respective paradigm.

## 3 Molecular principles of exercise and training adaptation

The repeated disruption of muscle homeostasis induced by each exercise bout engages adaptive processes that prime the muscle for subsequent challenges. This adaptive response, e.g. reflected in reduced symptoms of exercise-induced muscle damage and soreness [84,85], underscores the increased resilience and likely enhanced repair and regenerative capacity of trained muscle. This phenomenon is known as the “repeated bout effect” [86]. Even though the molecular underpinnings of muscle plasticity to individual and repeated contractions are only rudimentarily understood, general principles of adaptation in regard to epigenetic, transcriptomic, proteomic and functional changes have been proposed [15,87–89]. Most of the contemporary models are based on the observation of gradual adaptation of skeletal muscle to recurring perturbations elicited by individual exercise bouts. Such a training habituation has been associated with a diminishing response, at least in the case of non-progressive or submaximal stimulation in which a given training load exerts relatively smaller perturbations on a trained, adapted muscle. It therefore has been proposed that transcriptional events likewise follow such an attenuated pattern, with a lower amplitude of gene expression being found in trained compared to untrained muscle after an acute exercise bout [79,87,90]. Over time, the basal expression of genes encoding proteins important for the functional training adaptations was postulated to rise, thereby promoting muscle performance. However, such models are based on limited data and fail to comprehensively describe the complexity of the qualitative and quantitative changes elicited by training. For example, to a large extent, diminishing transcriptional regulation was based on the expression of PGC-1α after 7 HIIT exercise bouts [79]. However, in contrast, the other 8 genes tested in this study did not show such a pattern [79]. Moreover, an exacerbation of PGC-1α gene expression after an acute bout of endurance exercise in trained compared to untrained muscle has been reported in other studies, potentially influenced by the different exercise paradigms (HITT vs. one-legged knee extensor exercise), volume (7 vs. 20 sessions) and time points (4 and 24 vs. 0, 2, 6 and 24 h post-exercise) used [79,80]. Contemporary studies using –omics techniques have helped to shed more light on such discrepancies and reveal a more complex and nuanced picture of the response of untrained and trained muscle to an exercise bout. Exercise intensity, modality and training status belong to some of the strongest parameters that affect the outcome. With regard to intensity, we will discriminate between submaximal and exhaustion exercise. Even though HIIT or 9x8 repetitions at 80 % 1RM are very demanding, it is not considered exercise to exhaustion if it is not stated in such a way in the methods section. Examples for exercise to exhaustion include, but are not limited to, all-out sprints, time trials or resistance exercise to repetition failure.

Thus, whole genome transcriptomic and proteomic analyses now provide a more comprehensive picture of the molecular events that characterize the response of a training-naïve and a trained muscle to an acute exercise bout compared with the historic targeted analysis of individual genes. In the following sections, we will describe the current data and summarize these findings in an updated model of exercise response and training adaptation of skeletal muscle. Quantitative and qualitative differences in the acute exercise response in untrained and trained muscle will be discussed, including variations observed with distinct training paradigms and exercise intensities. Of note, the bulk of the findings originate from endurance exercise studies. Wherever possible, we have tried to juxtapose these to results obtained in resistance training.

### 3.1 Molecular signature of a trained muscle at rest

**Fig. 4:**
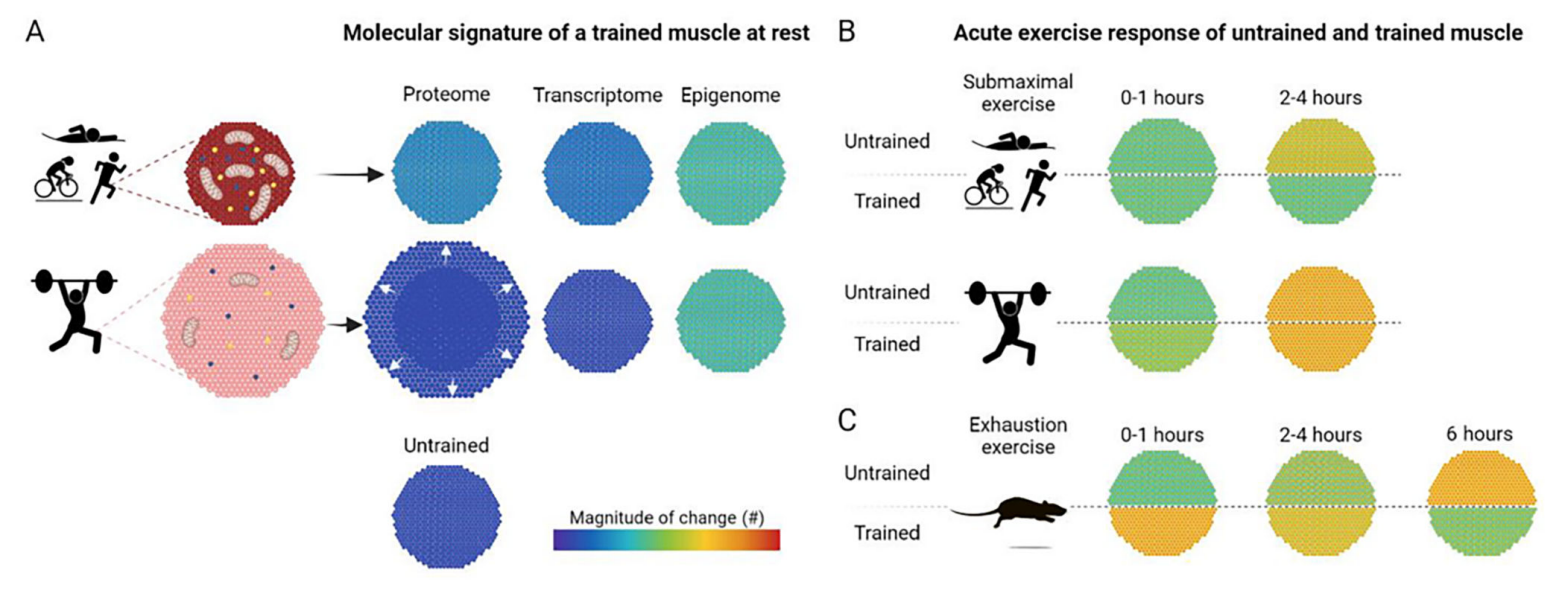
Molecular signature of a trained muscle at rest, and of untrained and trained muscles after an acute bout of endurance or resistance exercise. A.) Endurance- and resistance-trained muscles exhibit large differences in morphology, metabolism and function that contribute to the elevated oxidative metabolism or force generating capacity in these athletes, respectively. In endurance athletes, muscles at rest are characterized by a number of proteins that are different compared to untrained muscle. In contrast, little qualitative changes differentiate trained from untrained muscle in resistance exercise, suggesting that quantitative modulations of protein levels are the primary driver of training adaptation in the absence of relative alterations of cellular composition. Intriguingly, a surprisingly small number of genes exhibit changes in expression when comparing untrained and trained muscles at rest in either exercise modality. The largest differences at rest are observed in the number of differentially methylated regions in both, endurance- and resistance-trained muscle, suggesting a training state-specific response to an acute perturbation. B.) Gene regulation after an acute bout of submaximal endurance or resistance exercise in untrained and trained muscle. In submaximal exercise, the number of regulated genes is only attenuated in trained muscle in endurance, but not in resistance exercise. C.) In contrast to the response to submaximal exercise, the transcriptional regulation of untrained and trained mouse muscle is differentiated by a faster response, and an absence of attenuation in the trained compared to the untrained. Created with BioRender.com; sports icons from Adobe Stock, with permission.

The recurrent perturbations in response to repeated single exercise bouts over an extended duration (weeks to months) provoke a plethora of persistent adaptations that endure between individual exercise bouts and represent an “at rest” condition. The ensuing enhancements in muscle function, characterized by elevated force generation or fatigue resistance following resistance or endurance training, respectively, originate from improved neuromuscular control, muscle hypertrophy, increased vascularization, mitochondrial density, metabolic alterations and other plastic changes. Accordingly, the proteome of trained muscle substantially diverges from that of untrained muscle [8,22]. Intriguingly, endurance exercise elicits more pronounced remodeling of the muscle proteome compared to resistance training in muscles at rest, as evidenced by the markedly higher number of proteins changed in trained endurance athletes (Fig. 4A) [8]. More specifically, between 700 and 900 proteins were altered in endurance-trained female and male athletes, while only nine proteins were different in strength-trained athletes [8]. It however is conceivable that the extent of proteomic changes in resistance training, hallmarked by higher protein synthesis rates, is underappreciated: fiber hypertrophy could be primarily driven by quantitative changes of basally expressed proteins, which would not be discovered in mass spectrometry if equal amounts of protein from untrained and trained muscles are loaded. In contrast, there is a more pronounced alteration in the cellular composition in endurance trained muscle rather than changes in total muscle mass, resulting in a larger number of distinct proteins. For example, in line with the elevated oxidative capacity in endurance-trained individuals, a large proportion of the altered proteins are involved in oxidative phosphorylation (OXPHOS) and the tricarboxylic acid (TCA) cycle [8]. Strikingly, adaptations in proteins involved in aerobic respiration already manifest after 4 weeks of training in mice or 5 weeks of HIIT in humans [22,91]. Even faster, mitochondrial DNA (mtDNA) and CS activity are elevated after only 2 weeks of HIIT [79].

The extensive proteome remodeling observed in muscles of endurance athletes at rest suggests the presence of a distinct molecular signature indicative of the training status. Indeed, greater transcriptional changes are likewise found in endurance compared to those observed in strength athletes (Fig. 4A) [9,20]. Surprisingly, however, the changes at the transcriptional level are substantially fewer compared to those observe at the protein level [22,92]. In fact, less than 2 % of the transcriptome is changed in trained muscle at rest following a few weeks of moderate continuous training or HIIT in previously untrained humans and mice [22,93,94]. Similarly, a comparable low number of genes are altered in well-trained (>5 h of endurance exercise per week for at least 5 years) compared to untrained individuals at rest [95]. Of note, some studies report higher gene expression changes, which could potentially be attributed to more rigorous exercise paradigms, the investigation of athletes participating in a particular sport for at least 8 years, the use of less stringent statistical cut-offs, or other experimental discrepancies [9, 96,97]. Nevertheless, even in these studies with more transcriptional alterations in a trained muscle, the majority of transcripts exhibit only minor changes, typically with fold-changes below 1.5 [9,98]. Therefore, a trained muscle at rest does not differentiate from an untrained muscle by a large transcriptomic fingerprint. Functionally, genes altered in an endurance-trained muscle at rest are linked to energy metabolism, cellular respiration and slow-twitch muscle fibers, while the transcriptome of resistance-trained muscles exhibit differences in ECM remodeling [18,22,95]. Despite the overall functional similarity of the proteome and transcriptome, there is minimal overlap between the altered proteins and the regulated genes in a trained muscle [8,22,90].

Thus, a persistent change in basal gene expression mediating the long-term changes in the trained muscle proteome, as postulated in contemporary models [87], is not typically observed. The disconnect between gene expression and protein levels imply a more complex interaction, potentially involving post-translational modifications, protein stability, as well as additional means of transcriptional support as discussed below.

In contrast to the modest changes observed in the transcriptome of trained muscle at rest, distinct epigenetic profiles following both endurance and resistance training modalities are observed (Fig. 4A) [22, 99,100]. More specifically, trained muscles exhibit remarkable divergence with regard to differentially methylated genomic regions, which could prime the transcriptional response of the muscle for recurrent perturbations [22]. Once established, these epigenetic marks are relatively stable, and differ from the transient epigenetic modifications elicited by an acute exercise bout [15,22,99,101]. It has been hypothesized that such epigenetic modifications also contribute to “muscle memory”, the accelerated training progress following a period of detraining [102]. The temporal persistence in detraining, i.e. the absence of exercise bouts, and the overall contribution to muscle memory are however unclear. Moreover, training modality-dependent specificity exists [102]: endurance exercise studies have primarily focused on very long detraining periods (e.g. 9 months), failing to demonstrate enhanced improvements in performance or CS activity with prior training [103]. In contrast, evidence for muscle memory is more compelling in resistance exercise, where a greater increase in muscle strength was reported after 7 weeks of detraining, accompanied by an altered epigenetic landscape [99].

In summary, while at a transcriptional level, a trained muscle at rest does not exhibit substantial differences compared to an untrained muscle, functionally, morphologically, and epigenetically, these muscles are markedly divergent [10,22]. It therefore is conceivable that, based on the training-induced phenotypic adaptations, the molecular response of an untrained and a trained muscle to an acute bout of exercise is distinct.

### 3.2 Quantitative differences between the acute response in trained and untrained muscle to submaximal exercise

**Fig. 5:**
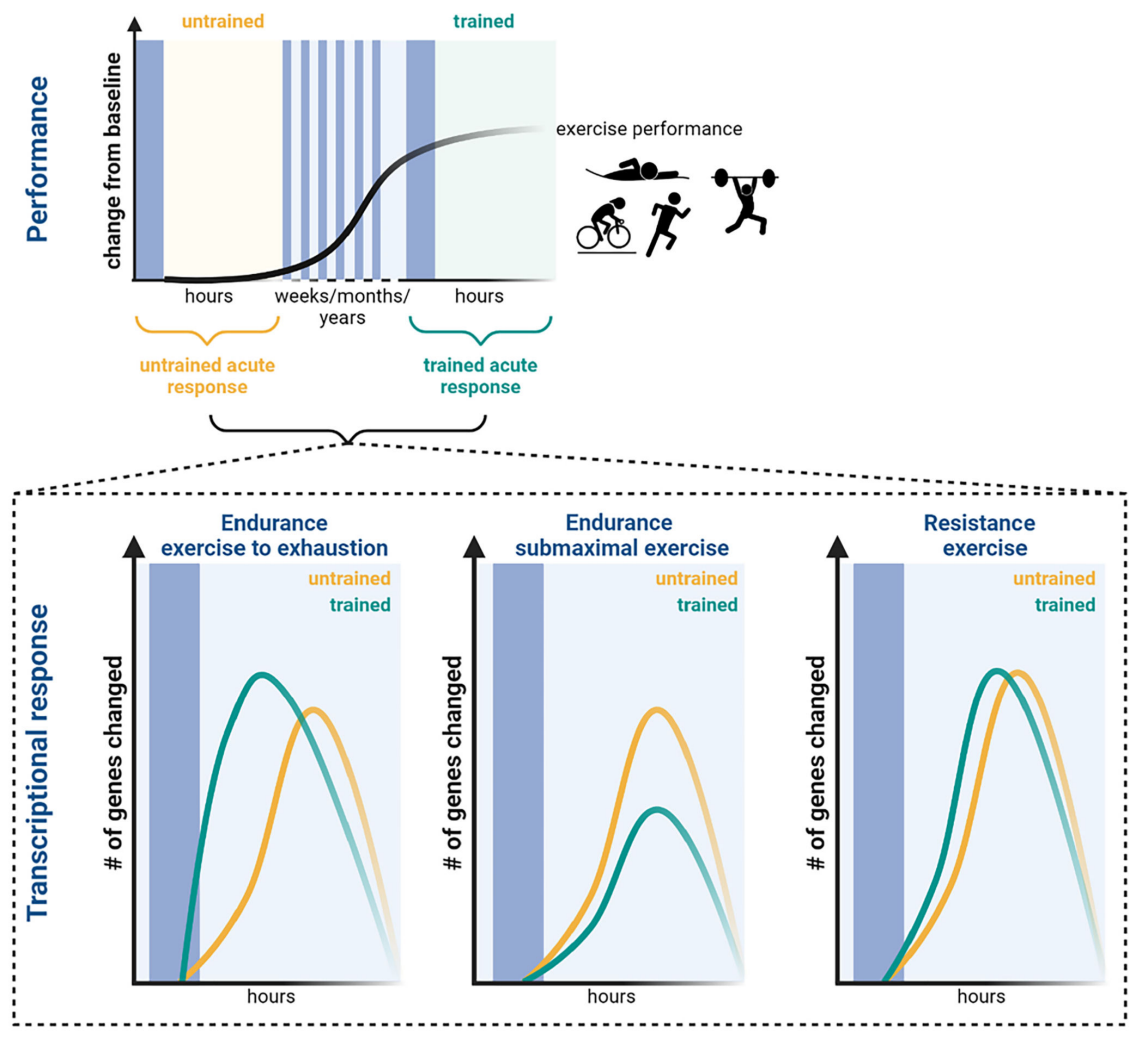
Temporal trajectories of the transcriptional response of untrained and trained muscle to a single bout of endurance or resistance exercise. The recurrent perturbations induced after single exercise bouts (depicted with the blue bars) repeated over weeks and month result in a pronounced training adaptation and increase in performance. Strikingly, the acute transcriptional response to the first bout of exercise is different compared to that of a trained muscle in regard to the number of genes, as well as amplitude and dynamics of regulation. Only submaximal endurance exercise elicits a lower transcriptional response in trained muscle (regarding the number of regulated genes) while this phenomenon is not observed after exhaustion endurance exercise or resistance exercise. In these contexts, the trained muscle even responds faster to the perturbation compared to untrained muscle. Created with BioRender.com; sports icons from Adobe Stock, with permission.

In terms of the early response to submaximal exercise (up to 3 h), the most notable transcriptional changes occur 3 h post-exercise, irrespective of the exercise paradigm or training status [20]. The number of regulated genes elicited by an acute endurance exercise bout performed at the same absolute intensity is diminished following a two weeks training period [68]. Similarly, endurance athletes exhibit less transcriptional changes after submaximal exercise $\left(30\min\text{at}\;75\%\dot{V}\;O_{2max}\right)$ compared to untrained individuals (Figs. 4B & 5) [20]. These data suggest that the perturbation to a submaximal endurance exercise bout is attenuated in trained individuals, resulting in fewer transcriptional changes. Even when untrained and endurance-trained individuals exercise at the same relative intensity, the metabolic challenge differs. For example, fractional utilization (i.e. lactate threshold, critical power) increases with training, indicating that perceived intensity at the same percentage $\dot{V}\;O_{2max}$ is higher in untrained compared to trained individuals, leading to a greater perturbation. Accordingly, blood lactate levels rises at approximately 65 % $\dot{V}\;O_{2max}$ in untrained individuals, while they remain low in trained individuals at the same relative intensity [104]. Furthermore, the increase in AMPK activity post-exercise that is observed in training-naïve individuals is blunted in endurance athletes [64,104]. This could be attributed to the lower metabolic stress elicited in endurance athletes at this intensity, or to the higher levels of muscle glycogen, both at rest and post-exercise, exerting an enhanced inhibitory effect on AMPK in endurance-trained individuals. As a consequence, endurance exercise at submaximal intensities might induce less perturbations in endurance-trained compared to untrained individuals, resulting in a diminished transcriptional response in term of the number of differentially regulated genes. This phenomenon is evident not only in well-trained athletes, but also observed in untrained individuals after 3 weeks of HIIT [93].

Intriguingly, this attenuated transcriptional response observed in trained muscle following acute endurance exercise did not manifest in the context of acute resistance exercise (Figs. 4B & 5). Specifically, one bout of knee extension exercise (9x8 repetitions at 80 % 1RM) induces similar or slightly larger transcriptional alterations after 3 h in strength athletes compared to untrained individuals [20]. Likewise, in a resistance-type exercise using nerve stimulation, which elicits hypertrophy as well as a fiber type shift and increase mitochondrial activity, the number of genes regulated 1 h post-exercise remains relatively consistent after 2, 10, 20 and 30 days of exercise [105]. It is noteworthy that the number of downregulated genes increases as training progresses, suggesting an explicit repression of certain genes that may contribute to the specificity of training adaptation [105]. Moreover, the temporal trajectory of gene expression appears to shift with training progression (Fig. 5). For example, strength athletes respond faster to a single bout of resistance exercise, as evidenced by 1.7 fold higher number of altered genes after 1 h when compared to untrained individuals [20]. The fact that the transcriptional response in strength athletes is not attenuated (in terms of the number of regulated genes) after resistance exercise suggests that even submaximal resistance exercise intensities induce substantial perturbations in trained muscle.

Besides the absolute number of genes, contemporary models have implied that the amplitude of the regulated genes is attenuated in trained individuals [15,79,87–89]. Transcriptome-based techniques now allow a broader exploration of this hypothesis, surpassing the predictive power of measurement of individual transcripts. In a 3 weeks HIIT paradigm, a correlation plot revealed a slightly lower amplitude of common regulated genes after the 9th bout compared to the 1st bout, even though the statistical significance of the magnitude of this effect is unclear [93]. In fact, among the 84 enriched pathways identified after acute exercise in untrained muscle, only four display reduced regulation in trained muscle [93]. Likewise, less than 10 % of the upregulated genes 3 h post-endurance exercise in untrained individuals are significantly higher compared to their counterparts in endurance-trained athletes [20]. This number is even lower with regard to the downregulated genes or in the context of resistance exercise [20]. Accordingly, the analysis of temporal trajectory patterns of genes regulated in untrained individuals reveal that the majority of these genes exhibit very similar behavior as in athletes [20]. There are subset of genes of which expression is amplified or attenuated in endurance or strength athletes compared to exercise-naïve individuals [20]. This transcriptional diversification suggests that athletes demonstrate an adapted response to recurrent exercise stimuli. Of note, many genes exhibit reduced variability in expression in trained compared to untrained muscles following an acute challenge [93]. Collectively, the current model suggesting an attenuated transcriptional response in trained individuals is oversimplified, and transcriptomic data are indicative of a more complex scenario involving dampening, exacerbation and unchanged amplitudes. In summary, submaximal exercise bouts elicit fewer transcriptional changes in terms of the number of genes rather than dampening of the amplitude of the regulated genes in trained compared to untrained muscle. However, training intensity might play a marked modulatory role.

### 3.3 Quantitative differences in trained and untrained muscle in response to an acute bout of exercise to exhaustion

The majority of studies investigating the transcriptional response of untrained and trained muscle have used submaximal exercise paradigms. Accordingly, far less is known about the transcriptional trajectories after exercise to exhaustion, which, in contrast to submaximal exercise, likely exerts similar cellular perturbations in untrained and trained muscle. We have made use of this advantage to investigate the molecular signature of an acute bout of exhaustion exercise in untrained and trained mice [22]. First, the total number of upregulated genes after exhaustion exercise in untrained and trained muscle after 0, 4, 6, and 8 h is similar [22], unlike the reduced number of genes altered after submaximal exercise in trained compared to untrained individuals [20,93]. Second, a substantial shift in the temporal trajectory of transcriptional induction from 6 h in the untrained to earlier time points in the trained muscle was observed (Figs. 4C & 5) [22]. These findings indicate a priming of trained muscle to react faster to an acute perturbation, coupled to a mitigation of the upregulation of genes at later time points. Third, the amplitude of change is very similar in the majority (~75 %) of the commonly upregulated genes [22]. Fourth, a substantially greater number of genes is downregulated in trained muscle following an acute challenge compared to untrained muscle [22]. In line with our observations, a bigger number of transcripts was also suppressed with training progression in resistance-like exercise in rats [105]. Furthermore, resistance exercise to failure induced a massive transcriptional response in trained individuals, resulting in a comparable number of up- and downregulated genes [83]. However, without a corresponding untrained cohort, the ability to draw conclusions about training status-dependent effects are limited in this study. In summary, in contrast to submaximal exercise, the transcriptional response is not attenuated in trained muscle when exercise is performed to exhaustion, neither in terms of absolute number of genes, nor regarding amplitude of change. In fact, the total response of the trained seems larger than that of the untrained muscle, in particular for downregulated gene transcription. The faster response of trained muscle to an acute exercise bout furthermore can be correlated with epigenetic marks that are seen in trained muscle at rest, implying a persistent priming compared to untrained muscle [22].

### 3.4 Qualitative differences in the acute exercise response of trained and untrained muscle

Interestingly, the quantitative and temporal differences are complemented by qualitative distinctions of the responses of untrained and trained muscle to an acute exercise bout. While the acute transcriptional response is very similar before and after 3 weeks of HIIT [93], the response in well-trained athletes is more distinct. In fact, only ~55 % of the upregulated genes in untrained individuals are also regulated in endurance-trained athletes (Fig. 3) [20]. Thus, around 45 % of the gene induction events are specific to the naïve exercise response. This training status-dependent specification is even more pronounced for suppressed genes, with only 30 % of the downregulated genes in untrained individuals also being regulated in athletes [20]. The diversification is larger if exercise bouts are performed to exhaustion, at least in rodent models (Fig. 3) [22]. In this case, only one-third of all upregulated genes are shared between untrained and trained muscle, while one-third is specific to the untrained and another third to the trained state [22]. Similar to human data, the extent of stratification is bigger for the transcriptional downregulation [22]. Intriguingly, the acute response to resistance exercise appears to be less determined by training status [20]. Approximately 75 % of the upregulated and 60 % of the downregulated genes in untrained individuals are also regulated in strength athletes (Fig. 3) [20]. Collectively, these findings indicate a marked specialization of trained muscle, in particular in endurance paradigms, leading to a large shift in the transcriptional response evoked by an acute exercise bout, indicative of an altered sensing, interpretation and reaction to the corresponding perturbations even in exercise to fatigue, in which the cellular perturbation should be comparable to that in the untrained counterpart.

## 4 The multicellular response of skeletal muscle to an acute exercise bout and chronic training

Novel single cell and single nucleus RNA sequencing (scRNA-seq and snRNA-seq, respectively) technologies provide opportunities to dissect the complex multicellular nature of tissues, facilitating the characterization of rare and poorly understood cell populations [106,107]. Skeletal muscle exhibits considerable heterogeneity, comprising diverse fiber types with distinct metabolic and contractile properties, as well as numerous tissue-resident cell types essential for proper muscle function [107]. In fact, only 40–60 % of nuclei in skeletal muscle originate from muscle fibers, at least in mice, with a higher proportion in fast-twitch/glycolytic compared to slow-twitch/oxidative muscles [108]. Non-myofiber nuclei originate from at least 10 distinct cell types, often comprised of numerous subpopulations [107–113]. Traditional bulk RNA-seq analyses fail to distinguish the response of the different cell types to exercise in muscle. It however has become evident that many of these tissue-resident cells play critical roles in the adaptive process. For example, endurance training increases the relative proportion of interleukin 13 (IL-13)-producing tissue-resident type 2 innate lymphoid cells, instrumental for the training-induced oxidative muscle phenotype [114]. In addition, regulatory T cells play an important role in physiological training adaptation of the muscle including endurance performance as well as mitochondrial morphology and function [115]. Moreover, exercise can elicit shifts within specific cell populations, e.g. in capillary muscle endothelial cells towards an increase in subpopulations with distinct angiogenic potentials, thereby promoting tissue vascularization [116]. These findings underscore the importance of multicellular crosstalk in facilitating proper muscle function and training adaptation.

Deconvolution of bulk RNA-seq results with single cell or single nucleus data offers predictions into the cellular contribution to the overall response in gene expression. For example, in endurance training, genes associated with ECM remodeling and axon guidance are predicted to predominantly be expressed in tenocytes, fibro-adipogenic progenitors (FAPs) and glial cells, and only to a smaller extent in muscle fibers [22]. Of note, deconvolution analyses are only predictive, and significantly depend on the data that are used to assign transcripts from bulk approaches and thus might be limited, e.g. when only untrained single cell or single nucleus data is available to infer the cell type-specific response to an acute exercise bout or chronic training. To obtain *bona fide* experimental results, scRNA-seq and snRNA-seq approaches will have to be performed in all of these contexts. Indeed, various findings have already been reported that allude to the multicellular response of muscle tissue. For example, among the tissue-resident cells, the transcriptomes of endothelial cells, smooth muscle cell and monocytes exhibit the most pronounced changes in a trained muscle at rest [117]. In contrast, acute all-out sprint exercise elicits the most substantial transcriptional changes in mesenchymal cells (likely FAPs) after 3 h, characterized by an enrichment of genes linked to regeneration and wound healing, followed by gene regulation in endothelial cells [118]. Surprisingly, only 25 % of the regulated genes in the scRNA-seq analysis are also identified as regulated in bulk RNA-seq data [118]. This discrepancy highlights some of the challenges imposed on deconvolution of bulk RNA-seq results, as well as possible drawbacks of bulk and scRNA-seq approaches. For example, bulk data could be compromised by low abundance of transcripts in few cells, or the counter-regulation of genes in different cell types. Moreover, scRNA-seq techniques of skeletal muscle harbor limited (or no) information on the large muscle fibers, and often impose undue activation of stress pathways during cell dissociation. Accordingly, scRNA-seq analyses reveal high levels of immediate early genes in unperturbed satellite cells, suggesting that conventional dissociation protocols induce a stress response in these cells [119]. Of note, the expression of these genes is not detected in cryosections using single-molecule RNA fluorescence *in situ* hybridization [119]. This issue has furthermore been highlighted by snRNA-seq of intact and dissociated muscles, resulting in clearly separated nuclear populations with a large number of differentially expressed genes [120]. Therefore, the study of the acute response of different muscle-resident cells to exercise, training or other cellular perturbations can be confounded by the activation induced by the dissociation protocol, including the transcription of immediate early genes and heat shock proteins. At least to some extent, these issues can be overcome with snRNA-seq, which therefore may be preferred for analyzing the acute exercise response. In addition to mitigating the dissociation bias, snRNA-seq allows the concomitant inclusion of myonuclei alongside with nuclei from mono-nucleated cells. Not surprisingly, different results have been obtained in studies using scRNA- or snRNA-seq. For example, a submaximal bout of exercise $\left(40\min\;\text{at}\;70\text{\%}\;\text{of}\dot{V}\;O_{2max}\right)$ induced the most pronounced transcriptional changes in myonuclei and a FAP subpopulation expressing Lumican (LUM+) [121]. Intriguingly, the genes regulated in FAPs are associated with axon guidance [121], in line with deconvolution predictions of bulk RNA-seq data [22]. Importantly, snRNA-seq experiments now for the first time enable a detailed analysis of different myonuclear populations. For example, snRNA-seq-based findings indicate that distinct myonuclear populations respond differently to an exercise stimulus [121]. Specifically, acute endurance exercise elicits more transcriptional changes in fast compared to slow muscle fiber myonuclei, with distinct outcomes in terms of gene expression, deviating from the association with energy metabolism characterizing commonly altered genes [121]. Second, even within the clusters of slow or fast muscle fiber myonuclei, a transcriptional divergence is observed between myonuclei from perturbed (post-exercise) and unperturbed muscle [121]. To date, a systematic and comprehensive analysis of the multicellular response of skeletal muscle to an acute exercise bout and chronic training is missing. Future studies leveraging these powerful techniques will reveal a much more fine-grained insight into the cellular crosstalk and molecular underpinnings of exercise perturbations and training adaptation. Such data might furthermore be combined with results from other tissues and organs, leading to a systems-wide atlas of the effects of exercise, analogous to the bulk analyses that have already been performed [122–124].

## 5 Summary, conclusions and future perspectives

**Fig. 6:**
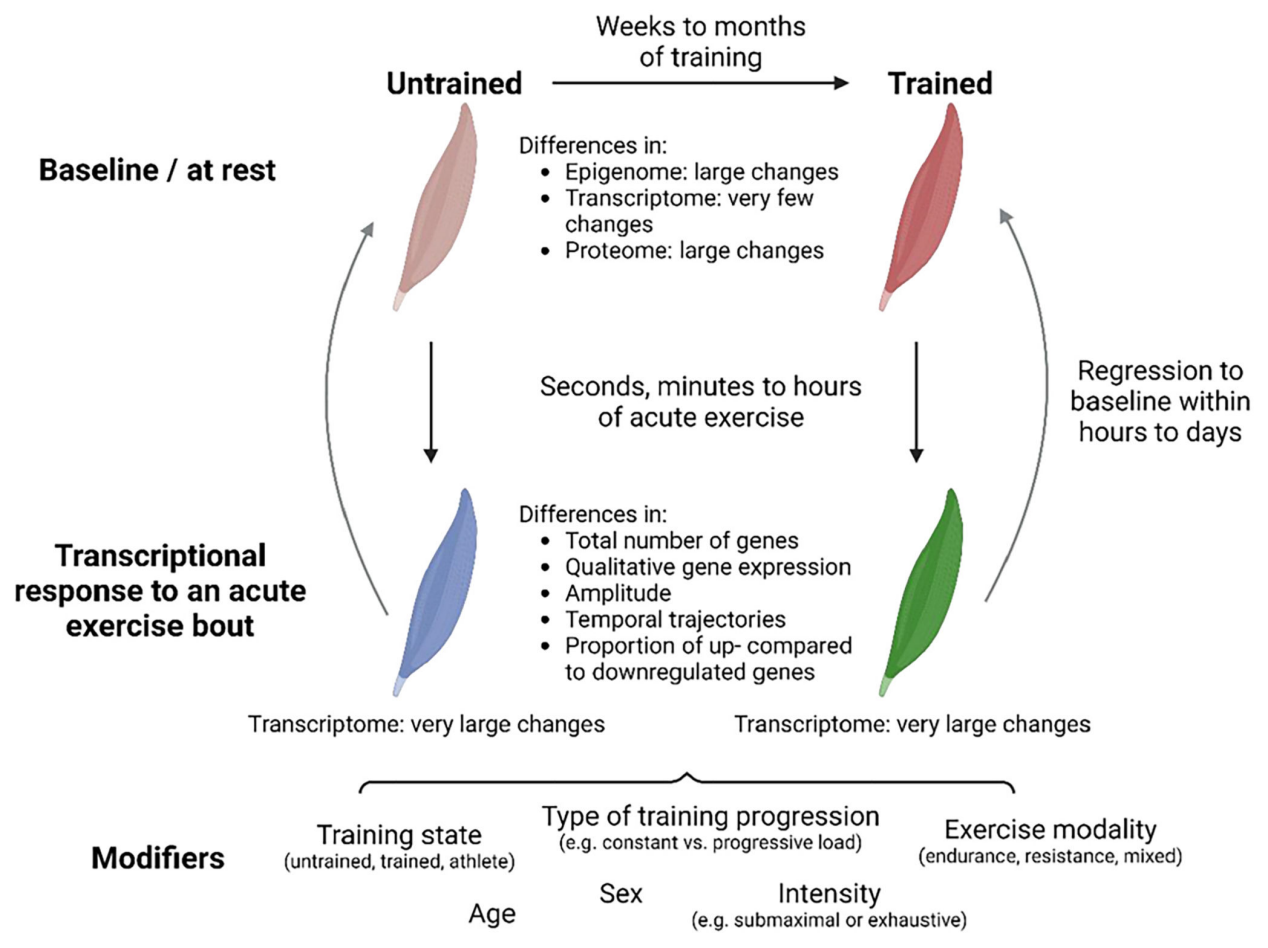
Molecular principles of exercise and training adaptation in skeletal muscle. A trained state at baseline/rest, evoked by repeated bouts of exercise over weeks to months, is primarily defined by a substantial modulation in the proteome, the functional basis of the cellular, morphological and functional adaptations that underlie improved performance. In contrast, surprisingly few persistent alterations in gene expression are observed in this context. However, the marked epigenetic remodeling indicates an altered, “primed” state, specifically directing the transcriptional response in the context of an acute perturbation. Indeed, an acute bout of exercise, performed over seconds to minutes to hours, results in the regulation of expression of a large number of genes, albeit with significant differences between untrained and trained muscles, e.g. in terms of quantitative aspects (number of genes, absolute amplitude of peak expression, at which time point after exercise, and which direction, i.e. induction or repression) and qualitative (which transcripts) aspects. In most cases, the strong transcriptional response to an acute bout of exercise will regress to baseline within hours to days, with significant variations between individual genes. The different colors of the muscle represent the extent of the difference between the conditions. The response of skeletal muscle to exercise is modified by many parameters, for example training state, training modality, intensity, training strategies, age or sex. Created with BioRender.com.

In the last decades, great strides have been made to better understand how skeletal muscles react to perturbations, in particular those elicited by exercise. Nevertheless, as outlined in this review, we are still far from understanding the molecular mechanisms that control these processes. As a consequence, the scientific foundation of training paradigms often still remains very thin, and strategies largely based on coach/athlete-derived lore. According to available and emerging data, it is clear that the molecular biology of skeletal muscle plasticity is far more complex and multifaceted than implied by contemporary models. First, for most of the molecular sensors, regulators and effectors, training paradigm specificity is unclear, and the underpinnings of the integration of the most often transient engagement leading to long-term training adaptations are unknown. For example, activation of mTORC1 has been reported in endurance and resistance training. Nevertheless, the consequence of inhibition, e.g. by rapamycin, has primarily been tested in resistance trials [125], even though a high probability of involvement in endurance training adaptations, in particular vascularization [126], exists. Second, the global principles that describe training adaptation surpass the simplistic models of exercise habituation-induced attenuation over time. In fact, marked qualitative and quantitative changes in gene expression are observed, with a substantial number of transcript found at higher levels in trained compared to untrained muscle after an acute exercise bout and *vice versa*. Factors that markedly alter this process include training intensity (e.g. submaximal or exhaustive), load (constant or progressive), modality (endurance, resistance or mixed), training state, sex, age, as well as other parameters (Fig. 6). Moreover, the type of muscle (fast-twitch/glycolytic or slow-twitch/oxidative, degree of functional involvement in movement), and the time of biopsy in the acute exercise bout setting (different time points during and post-exercise) as well as the chronic training context (*bona fide* at rest situation, unperturbed by the last acute exercise bout and detraining) have a significant impact on the outcome. To overcome some of these conceptual hurdles, a broader view on the molecular mechanisms that control exercise adaptation should be adopted. To do so, entrenched “science lore”, in which the “usual suspects” are studied in a limited context, e.g. PGC-1α or AMPK in endurance, or mTORC1 in resistance training, despite ample evidence of a much broader engagement, has to be avoided. Third, attention should be paid to a precise and detailed description of the experimental design, using multiparametric descriptors of participants and training protocols to allow a better comparison and potential integration of different studies. Timing is of obvious importance leading to vastly different results in mice [22] and humans [127]. Moreover, since human studies rely on small biopsies, most often obtained from the *Musculus vastus lateralis*, variation can be introduced by the sampling location [128,129] as well as confounding effects of repeated sampling [130–132]. Fourth, a common understanding on how experimental design affects interpretation should be established, e.g. in terms of the study of acute exercise bouts, chronic training adaptations, at rest/unperturbed states and training state-effects. For example, data on trained individuals often are confounded by persisting effects of the last exercise bout. Such effects can linger for several days, e.g. muscle weakness 3 days after endurance exercise in untrained volunteers [133], or even in trained individuals, e. g. as implied by the elevated creatine kinase levels 36 h after the last resistance exercise bout [134]. The exact window representing an at-rest state remains to be determined, and the events leading to detraining better elucidated, first signs of which have been reported at 1 week post-exercise, initiating a process lasting over weeks to months [135, 136]. Then, more comparative studies of model organisms and humans might help to unravel the mechanistic details of muscle plasticity. Even though the relevance of data obtained in mice, rats and other models in regard to human biology needs to be constantly validated, studies of these models provide several advantages over human investigations, in particular to disentangle causality from epiphenomena (Table 1). Finally, scRNA-seq and snRNA-seq experiments, coupled to spatial techniques, will be instrumental to better understand the dynamics of multicellular crosstalk in muscle tissue in exercise and other contexts. Inversely, more systematic investigation of the global effects of exercise on other tissues and organs will help to understand how physical activity affects the whole body. Groundbreaking work by the Molecular Transducers of Physical Activity Consortium (MoTrPAC) and others already now provide a blueprint and data resources to study this on a systems level in more detail [122]. The use of multi-omics technologies will thereby help to integrate epigenetic, transcriptomic, proteomic, phosphoproteomic and other data [124]. For example, a temporal analysis of multi-omics provides potential explanations for the apparent disconnect between transcript and proteins that have been reported, mostly in single time-point experimental designs [90]. Integrated studies have demonstrated a good correlation between the changes in the proteome in endurance trained muscle at rest, and the transcripts that are regulated after an acute exercise bout in untrained or trained muscle, combined with those modulated in trained muscle at rest [22]. These observations imply a complex regulatory landscape in which proteins are controlled by different modalities, e.g. by an integration of acute transcriptional regulation in each exercise bout that can differ between untrained and trained muscle.

In summary, even though a large number of sensors, regulators and effectors are known, our insights into the molecular mechanisms that control the response of skeletal muscle to an acute exercise bout remain poor, and even more so in regard to long-term training adaptations. Intriguingly, there is a large transcriptional overlap between the acute response to endurance and resistance exercise and it is unclear how training specificity is brought about (Fig. 3). It is conceivable that the transcriptional response is more specific as training progresses, as a trained muscle demonstrates a highly specialized response to acute challenges, with less transcriptional noise (Fig. 6). Of note, the suppressed genes after a single bout of exercise seems to be more specific to the exercise paradigm and training status compared to the upregulated genes. Hence, an agnostic view of the data implies a complex system, for which signal integration, interpretation and output are still mysterious, as are the underpinnings of training specification in different modalities. Importantly, even so-called master regulators are dispensable for some aspects of training adaptation, indicating a hitherto underappreciated robustness and adaptability of the system, even in unfavorable conditions [10,22]. It therefore might be of no surprise that in large meta-analyses of human exercise trials, many factors that have been entrenched in “training lore” seem of minor importance, e.g. the “intensity paradox” showing that endurance exercise exerts cardiorespiratory benefits regardless of intensity [137], or that lower and higher load resistance training can lead to comparable outcomes, at least for some parameters [138]. Similarly, basic aspects of training adaptation are still controversial, e.g. whether muscle growth contributes to the increase in strength in resistance training [139,140] and the implication of satellite cells in hypertrophy [141,142], which type of exercise is optimal for athletes [143,144], or whether intensity or volume of endurance training have a bigger effect on mitochondrial content [145,146]. Many of these controversies arise from the interpretation of studies with small participant numbers, specific training paradigms, and often small effect sizes, which then are extrapolated to formulate general principles. A more refined view, combined with better information about cause and effect, will hopefully help to design and implement science-based, personalized and safe training strategies with proven efficacy for patients, the general population and athletes.

## Figures and Tables

**Table 1:** Comparison of the advantages and disadvantages of exercise studies in humans and mice. Optimally, the respective strengths would be leveraged in combining the results from such investigations.

	Human exercise physiology	Mouse studies
**Human relevance**	Relevance for human biology	Translatability to human physiology?
**Study size**	Often small n numbers	Often small n numbers
**Study participants**	Often college-aged, moderately active, Caucasian males	Often only studied in males
**Heterogeneity**	Large heterogeneity (genes, epigenome)	Heterogeneity can be determined by the use of in- or outbred strains
**Variability**	Large variability (diet, sleep, non-exercise activated thermogenesis, chronotype, motivation, …)	Highly standardized conditions (diet, temperature, humidity, day-night cycles, activity levels, …)
**Exercise paradigms**	All relevant exercise paradigms available	Well established high- and low-stress endurance training protocols, few resistance training paradigms
**Study material**	Restricted access to muscle samples (biopsies), mostly obtained in one muscle (*M. vastus lateralis*): heterogeneity due to time and place of sampling, confounded by repeated sampling	Access to whole muscles and very different muscle beds
**Type of studies**	Descriptive or associative	Mechanistic, descriptive and associative due to the availability of genetic, pharmacological and other tools
